# Differentiation of RPE cells from integration-free iPS cells and their cell biological characterization

**DOI:** 10.1186/s13287-017-0652-9

**Published:** 2017-10-02

**Authors:** Roni A. Hazim, Saravanan Karumbayaram, Mei Jiang, Anupama Dimashkie, Vanda S. Lopes, Douran Li, Barry L. Burgess, Preethi Vijayaraj, Jackelyn A. Alva-Ornelas, Jerome A. Zack, Donald B. Kohn, Brigitte N. Gomperts, April D. Pyle, William E. Lowry, David S. Williams

**Affiliations:** 1 Stein Eye Institute and Department of Ophthalmology, David Geffen School of Medicine at UCLA, 100 Stein Plaza, Los Angeles, CA 90095 USA; 2 Department of Microbiology Immunology and Molecular Genetics, Los Angeles, CA USA; 3 Department of Molecular Cell and Developmental Biology, Los Angeles, CA USA; 4 Department of Pediatrics, David Geffen School of Medicine, Los Angeles, CA USA; 5 Eli and Edythe Broad Center of Regenerative Medicine and Stem Cell Research at UCLA, Los Angeles, CA USA; 6 Jonsson Comprehensive Cancer Center, Los Angeles, CA USA; 7 Department of Medicine, David Geffen School of Medicine, Los Angeles, CA USA; 8 Department of Population Sciences, City of Hope National Medical Center, Duarte, CA USA; 9 Department of Neurobiology, David Geffen School of Medicine, Los Angeles, CA USA; 10 Molecular Biology Institute, Los Angeles, CA USA; 11 Brain Research Institute, University of California, Los Angeles, CA USA

**Keywords:** Retinal pigment epithelium, Induced pluripotent stem cells, RPE cytoskeleton, Live-cell imaging, Phagocytosis

## Abstract

**Background:**

Dysfunction of the retinal pigment epithelium (RPE) is implicated in numerous forms of retinal degeneration. The readily accessible environment of the eye makes it particularly suitable for the transplantation of RPE cells, which can now be derived from autologous induced pluripotent stem cells (iPSCs), to treat retinal degeneration. For RPE transplantation to become feasible in the clinic, patient-specific somatic cells should be reprogrammed to iPSCs without the introduction of reprogramming genes into the genome of the host cell, and then subsequently differentiated into RPE cells that are well characterized for safety and functionality prior to transplantation.

**Methods:**

We have reprogrammed human dermal fibroblasts to iPSCs using nonintegrating RNA, and differentiated the iPSCs toward an RPE fate (iPSC-RPE), under Good Manufacturing Practice (GMP)-compatible conditions.

**Results:**

Using highly sensitive assays for cell polarity, structure, organelle trafficking, and function, we found that iPSC-RPE cells in culture exhibited key characteristics of native RPE. Importantly, we demonstrate for the first time with any stem cell-derived RPE cell that live cells are able to support dynamic organelle transport. This highly sensitive test is critical for RPE cells intended for transplantation, since defects in intracellular motility have been shown to promote RPE pathogenesis akin to that found in macular degeneration. To test their capabilities for in-vivo transplantation, we injected the iPSC-RPE cells into the subretinal space of a mouse model of retinal degeneration, and demonstrated that the transplanted cells are capable of rescuing lost RPE function.

**Conclusions:**

This report documents the successful generation, under GMP-compatible conditions, of human iPSC-RPE cells that possess specific characteristics of healthy RPE. The report adds to a growing literature on the utility of human iPSC-RPE cells for cell culture investigations on pathogenicity and for therapeutic transplantation, by corroborating findings of others, and providing important new information on essential RPE cell biological properties.

**Electronic supplementary material:**

The online version of this article (doi:10.1186/s13287-017-0652-9) contains supplementary material, which is available to authorized users.

## Background

The retinal pigment epithelium (RPE) is a monolayer of cells that provides essential roles for the function and viability of the photoreceptor cells [1]. Age-related macular degeneration (AMD) is a widespread and common disease among older people, leading to irreversible loss of central vision. The death of macular photoreceptors has been suggested to be secondary to the degeneration of the RPE [2–5]. Therefore, one promising form of treatment for AMD is the transplantation of healthy RPE cells into the retinas of human patients to restore lost functions, and potentially halt or reverse the progression of the disease. Pluripotent stem cells, including both human embryonic stem cells (ESCs) and human induced pluripotent stem cells (iPSCs), can provide a renewable source of human RPE cells, which are potentially amenable for studying normal and disease mechanisms in culture, and for intraocular transplantation for disease treatment. Patient-derived iPSC-RPE cells offer disease modeling and testing of pharmacologically active compounds, in addition to autologous transplantation, without the need for immunosuppression [6]. Originally, iPSCs were generated by exogenous expression of the factors described by Yamanaka’s group (OCT4, SOX2, KLF4, and c-MYC) from retroviral vectors, thus resulting in genome integration [7]. To circumvent the risks of genome integration, several nonintegrating methods are now being used to induce pluripotency in mammalian cells, including nonintegrating episomal vectors [8], delivery of RNA [9] and proteins [10], and use of small molecule compounds [11]. Once reprogrammed to pluripotency, the iPSCs can spontaneously differentiate along the neural lineage, and, further, to RPE cells, which are readily discernible due to their pigmentation and cobblestone appearance [12, 13]. Several laboratories have now published protocols for the differentiation of human ESCs or iPSCs to RPE cells, using more directed approaches so as to increase the yield of RPE cells [14–17]. Taken together, the current technology allows for the generation of patient-specific iPSCs that are free of integrated reprogramming genes, and can subsequently be used to generate the quantities of functional RPE cells necessary for transplantation purposes.

This paper reports the use of a nonintegrating approach to generate iPSCs for the generation of RPE cells under GMP-compatible practices. We have performed in-vitro characterization of the iPSC-RPE cells to test whether they express RPE-specific genes and proteins. Importantly, we have also tested for the first time whether RPE cells, derived from any type of stem cell, possess normal cytoskeletal organization, organelle motility, and phagosome ingestion with degradation kinetics, thus detailing critical cellular functions of the RPE in relation to RPE dystrophy. The importance of these tests, which includes live-cell imaging analysis, has been emphasized by recent studies showing that defects in intracellular motility lead to RPE pathogenesis like that in AMD [18], potentially the most significant target disease of RPE transplantation. We have also tested these iPSC-RPE cells in vivo, using mouse models, to determine whether the cells can integrate into a recipient tissue, and rescue a function lost by the host retina. Our results show that iPSCs, generated with a nonintegrating method, can serve as a renewable source of functional RPE cells, which can be used for detailed cell biological analyses of pathogenicity in vitro, as well as for transplantation in treatment of retinal diseases.

## Methods

### GMP facility

Fibroblast derivation, iPSC generation, and RPE differentiation were performed in a GMP-compatible facility at the Eli and Edythe Broad Center of Regenerative Medicine and Stem Cell Research at UCLA– California Institute for Regenerative Medicine Shared Research Laboratories as described previously [19]. All cells used in this study were handled by qualified personnel. GMP-compatible protocols and procedures were followed. The facility and equipment were routinely cleaned, calibrated, and monitored rigorously by contract vendors. All materials used were qualified according to the supplier certificate of analysis. Inventory records, and generation and distribution of materials, were documented.

### Derivation of fibroblasts

Pieces of skin biopsy (1 mm^2^) were incubated with 1 mg/ml AOF Collagenase A (LS00415; Worthington Biochemical) for 1 h. Released cells were washed twice and plated on dishes, coated with CellStart™ (A1014201; Gibco), in Fibrogro™ medium (SCM037; EMD Millipore). Once confluent, the cells were passaged using TrypLE™ Select (12563-011; Invitrogen), and their purity was determined by the proportion of cells expressing fibroblast cell markers. The cells were stable in culture for at least five passages.

### Generation and maintenance of iPSCs

The nonintegrating vector used for reprogramming of fibroblasts to iPSCs was the modified, noninfectious, self-replicating Venezuelan Equine Encephalitis (VEE) virus RNA replicon RNA system from EMD Millipore (catalog number SCR550). This synthetic polycistronic RNA replicon has all four reprogramming factors on a single RNA strand, thereby eliminating the need to transfect multiple individual mRNAs, and increasing the reprogramming efficiency over DNA-based and protein-based reprogramming methods. Briefly, fibroblasts were transfected with the vector, and selected with puromycin for 9–11 days in the presence of B18R protein (GF156; EMD Millipore). Removal of the B18R protein mediates the elimination of the RNA replicon system from the cultures. Selected cells were passaged onto Matrigel (354277; BD) and allowed to grow for 3–4 weeks, during which time iPSC colonies began to form. These colonies were picked and passaged to establish individual iPSC lines, which were subsequently maintained in culture under feeder-free conditions, using a 1:1 formulation of TeSR2 medium (05860; Stem Cell Technologies) and NutriStem® medium (01-0005; Stemgent). A bioinformatics assay for pluripotency, PluriTest, was performed by Cedars-Sinai. Karyotyping was performed by Cell Line Genetics.

### Differentiation of iPSCs into RPE cells

RPE cells were differentiated from iPSCs, with modifications of a method described previously [14]. The iPSCs were cultured for 2 weeks as embryoid bodies (EBs), suspended in low-adherent dishes in basal medium, containing Dulbecco’s Modified Eagle Medium: Nutrient Mixture F-12 (DMEM/F12) (11330-032; Invitrogen), supplemented with 14% xeno-free knockout serum (12618-013; Invitrogen), 0.1 mM nonessential amino acids (NEAA) (11140-050; Invitrogen), 2 mM GlutaMax™ (35050-061; Invitrogen), and 10 mM nicotinamide (N0636; Sigma-Aldrich). The medium was changed every other day. Growth factors including Activin A (140 ng/ml, 120-14P; Peprotech), transforming growth factor beta 1 (TGFB1) (2.5 ng/ml, AF-100-21C; Peprotech), and fibroblast growth factor 2 (FGF2) (20 ng/ml, 100-18B; Peprotech) were then added to the basal medium, and the EBs were allowed to grow and differentiate for an additional 2 weeks. The EBs were then returned to basal medium until pigmentation became evident. The pigmented regions in the EBs were separated by scalpel dissection, and plated as adherent cultures in RPE medium: DMEM/F12 with 5% fetal bovine serum (FBS) (FB-12; Omega Scientific), 4% normal human AB serum (IPLA-SERAB-HI; Innovative Research), triiodothyronine (0.02 ng/ml, T6397; Sigma-Aldrich), hydrocortisone (0.02 μg/ml, H0888; Sigma-Aldrich), taurine (0.25 mg/ml, T0625; Sigma-Aldrich), 10 mM nicotinamide, 0.1 mM NEAA, 1× N1 (N6530; Sigma-Aldrich), 1 × B27 (A14867-01; Invitrogen), 0.1 mM β-mercaptoethanol (M3148; Sigma-Aldrich), and GlutaMax™. Pigmented cells were passaged, following gentle collection with medium after 5-min TrypLE™ treatment. For the following analyses, RPE cells were cultured in RPE medium (as earlier, but lacking B27 and β-mercaptoethanol, and containing MEM alpha (32561-037; Invitrogen) with 1% FBS), at 37 °C and in 5% CO_2_. For all experiments, iPSC-RPE cells were passaged one to four times beyond their derivation from the pigmented EBs, and unless otherwise stated the results in the figures were obtained from iPSC-RPE line 2.

### Immunocytochemistry

iPSC-RPE cells were seeded (1.66 × 10^5^ cm^–2^) on Transwell inserts (3470; Corning) or 12-mm glass coverslips, coated with laminin (23017015; Thermo Fisher), and cultured for 6–8 weeks. Cultures were washed twice with phosphate-buffered saline (PBS), fixed with 4% formaldehyde in PBS for 10 min, washed with PBS, permeabilized with 0.25% Triton X-100, and then blocked with 4% bovine serum albumin (BSA) or 10% normal goat serum (NGS) (50-062Z; Invitrogen) in PBS for 1 h. They were immunolabeled for 1 h at room temperature in PBS, containing 1% BSA or 10% NGS, and antibodies against the following proteins: SOX2 (sc-17320; Santa Cruz Biotechnology), NANOG (AB9220; EMD Millipore), OCT4 (#2840; Cell Signaling Technology), fibroblast-specific protein-1 (FSP-1) (ABF32; EMD Millipore), vimentin (ab92547; Abcam), RPE65 (ab67042; Abcam), microphthalmia-associated transcription factor (MITF) (ab20663; Abcam), bestrophin 1 (BEST1) (ab2182; Abcam), zona occludens-1 (ZO-1) (40-2200; Life Technology), occludin (ab31721; Abcam), claudin19 (H00149461-M02; Novus Biologicals), rhodopsin (RHO) (RHO pAb01 [20, 21]), integrin α_V_β_5_ (ab24694; Abcam), and MER proto-oncogene, tyrosine kinase (MERTK) (NB110-57199; Novus Biologicals). Following primary antibody incubation, cultures were washed 3 × 5 min, and incubated with Alexa Fluor-conjugated secondary antibodies (Invitrogen) for 1 h at room temperature, in the dark. Cultures were washed 3 × 5 min with PBS. Membranes of Transwell inserts were excised and mounted onto frosted microscope slides using Fluoro-Gel II mounting medium with 4,6′-diamino-2-phenylindole (DAPI) (17985-50; Electron Microscopy Sciences) to counterstain the nuclei. Images were acquired with an Olympus FluoView 1000 confocal microscope or a Zeiss Axiovert 200 M microscope.

### Phagocytosis of photoreceptor outer segments

Porcine eyes were obtained from a local slaughterhouse for the purification of photoreceptor outer segments (POSs), following a method used previously for bovine POS purification [22]. Briefly, retinas were isolated and homogenized under dim red light. The homogenate was then loaded onto a continuous (27–50%) sucrose gradient to purify the POSs, which contain outer segments from rods (ROSs) and cones (COSs). The POSs were frozen in DMEM with 2.5% sucrose at – 80 °C. For phagocytosis assays, the POSs were thawed at room temperature and incubated with iPSC-RPE cells on laminin-coated Transwell inserts (10 POSs/cell) for 2 h. After the POS challenge, cells were washed with PBS, containing 0.9 mM calcium and 0.49 mM magnesium (PBS-CM), and immediately processed for immunofluorescence (pulse), or incubated further before processing for immunofluorescence (chase).

A double immunofluorescence labeling strategy, using an antibody against RHO, was used to distinguish between ROSs bound to the surface of the iPSC-RPE cells and ROSs that have been internalized, as described previously [23, 24]. Briefly, cultures were fixed with 4% formaldehyde for 10 min, and blocked with 1% BSA in PBS-CM for 15 min. Surface-bound ROSs were labeled with the RHO pAb01, followed by an Alexa Fluor 488-nm-conjugated goat anti-rabbit secondary antibody. After permeabilization with 50% ethanol in PBS-CM for 5 min, all ROSs were labeled with the same RHO antibody, followed by an Alexa Fluor 594-nm-conjugated goat anti-rabbit secondary antibody. Finally, cells were washed with DPBS-CM before the membranes of the Transwell inserts were excised and mounted onto microscopy slides. Confocal Z-stacks of randomly selected fields of view were acquired on an Olympus confocal microscope using a 60× NA1.4 oil objective. Surface-bound ROSs were labeled with both secondary antibodies, thereby appearing yellow. Internalized ROSs were labeled only with the Alexa Fluor 594-nm-conjugated secondary antibody, and therefore appeared red. For quantification, ROSs with a minimum diameter of 0.5 μm were counted from a total of six to eight fields of view using imageJ software. Analysis of ROS degradation was performed by comparing the total number of ROSs after the 2-h pulse with the number after 2-h and 5-h chase periods.

### Live-cell imaging

iPSC-RPE cells were plated on laminin-coated Lab-Tek™ chambered coverglass (155411; Fisher Scientific), and allowed to polarize for 8 weeks. To label acidic organelles, including endolysosomes, the cells were incubated with RPE medium containing 100 nM LysoTracker Red DND-99 (L7528; Thermo Scientific) for 1 h at 37 °C. After washing to remove excess dye, fresh medium containing 25 mM HEPES (15630-080; Gibco) was added to the cells. Live-cell imaging was performed using an Ultraview ERS spinning disk with a Zeiss Axio Observer microscope, and an environmental chamber maintained at 37 °C. Movies were acquired with a 63× oil objective at 1.9 frames per second, using Volocity (PerkinElmer). The trajectories of labeled organelles were analyzed in the *x* and *y* dimensions, during a time period of 20–40 s, using Volocity and Imaris × 64 (Bitplane) software.

### Transepithelial resistance measurements

Transepithelial resistance (TER) was measured for iPSC-RPE cells cultured on laminin-coated Transwell inserts (growth surface area, 0.33 cm^2^), using an EVOM^2^ Epithelial Voltohmmeter (World Precision Instruments) with a STX2 electrode. Measurements were made within 3 min of removal from the incubator. The net TER was determined by subtracting the resistance across a laminin-coated Transwell insert, lacking cells, from measured values, and then multiplying by the surface area.

### RNA preparation and expression analysis

Total RNA from the iPSC-derived RPE was extracted using the RNeasy Mini Kit (74104; Qiagen). RNA concentrations were measured using a Qubit fluorometer. Single-strand cDNA was synthesized from 200 ng of total RNA, using Superscript IV and random hexamer primers (N8080127; Fisher Scientific) in a volume of 20 μl. The cDNA was used for semi-quantitative reverse transcription-polymerase chain reaction (RT-PCR) analysis. PCR reactions were performed using GoTaq® Flexi DNA polymerase (M829; Promega). Thermal cycling conditions were performed as follows: one cycle at 94 °C for 300 s; 30 cycles at 94 °C for 30 s, 60 °C for 30 s, and 72 °C for 30 s; and one cycle at 72 °C for 300 s. The sequences of primers used for the PCR include: *RPE65*, 5′-TCCCCAATACAACTGCCACT-3′ and 5′-CCTTGGCATTCAGAATCAGG-3′; *MERTK*, 5′-TCCTTGGCCATCAGAAAAAG-3′ and 5′-CATTTGGGTGGCTGAAGTCT-3′; *BEST1*, 5′-TAGAACCATCAGCGCCGTC-3′ and 5′-TGAGTGTAGTGTGTATGTTGG-3′; and *GAPDH*, 5′-ACCACAGTCCATGCCATCAC-3′ and 5′-TCCACCACCCTGTTGCTGTA-3′ [25].

### Western blot analyses

Cells were lysed in RIPA-I lysis buffer (89900; Fisher Scientific) with added protease inhibitor cocktail (11836153001; Roche). Protein concentrations were estimated using a Qubit fluorometer (~30 μg protein was applied to each lane). Proteins were transferred to Immobilon PVDF membranes (IPVH00010; EMD Millipore), which were blocked with TBS with 0.05% Tween 20 (P9416; Sigma-Aldrich) and 5% skimmed milk for 30 min, and then probed with anti-RPE65. HRP-conjugated secondary antibodies were visualized by enhanced chemiluminescence (RPN2232; GE-Healthcare).

### Subretinal injections

iPSC-RPE cells were injected into the subretinal space of *Mertk* ^*–*/–^ mice (129 genetic background) and BALB/cJ albino mice. Cultures of iPSC-RPE cells were washed thoroughly with PBS before enzymatic dissociation with TrypLE™. BSS PLUS™ (0065080050; Alcon Laboratories) was added to create a suspension at a concentration of 50,000 cells/μl. Mice (postnatal day (P) 10–16) were anesthetized by isoflurane inhalation. Their pupils were dilated with a drop of 1% (w/v) Atropine Sulfate ophthalmic solution (17478-215-02; Akorn Pharmaceuticals), and the corneas were kept moist with Hypromellose ophthalmic demulcent 2.5% solution (51394-315-15; Wilson Ophthalmic Corp.). A 1-μl suspension of iPSC-RPE cells was injected into the subretinal space of each eye, under a Zeiss Stemi 2000 microscope, as described previously [26]. Ophthalmic ointment (Neomycin & Polymyxin B sulfates and Dexamethasone, 61314-631-36; Falcon Pharmaceuticals) was applied to each eye immediately following injection. Cyclosporine (200 mg/l, 0078-0109-61; Novartis) was added to the drinking water of the dam from 1 day prior to the injection until the pups were weaned at P28. Mice were kept on a 12-h dark/12-h light cycle. For experiments concerning phagosomes, they were killed between 15 and 30 min after lights on.

### Microscopy

For fluorescence microscopy, eyes were fixed, embedded in OCT, cryosectioned, and immunolabeled as described previously [18]. Semi-thin sections were prepared for light microscopy by fixation and Epon embedment, as described previously [18].

### Statistical test

GraphPad Prism 7 and Microsoft Excel were used to perform statistical analyses. The data were represented by the mean ± the standard deviation or standard error of the mean. A two-tailed Student *t* test was used to determine whether there was a significant difference in photoreceptor nuclei counts between control vs iPSC-RPE-injected eyes. *p* ≤ 0.05 was considered statistically significant.

## Results

### RNA-based reprogramming of fibroblasts into iPSCs

**Fig. 1:**
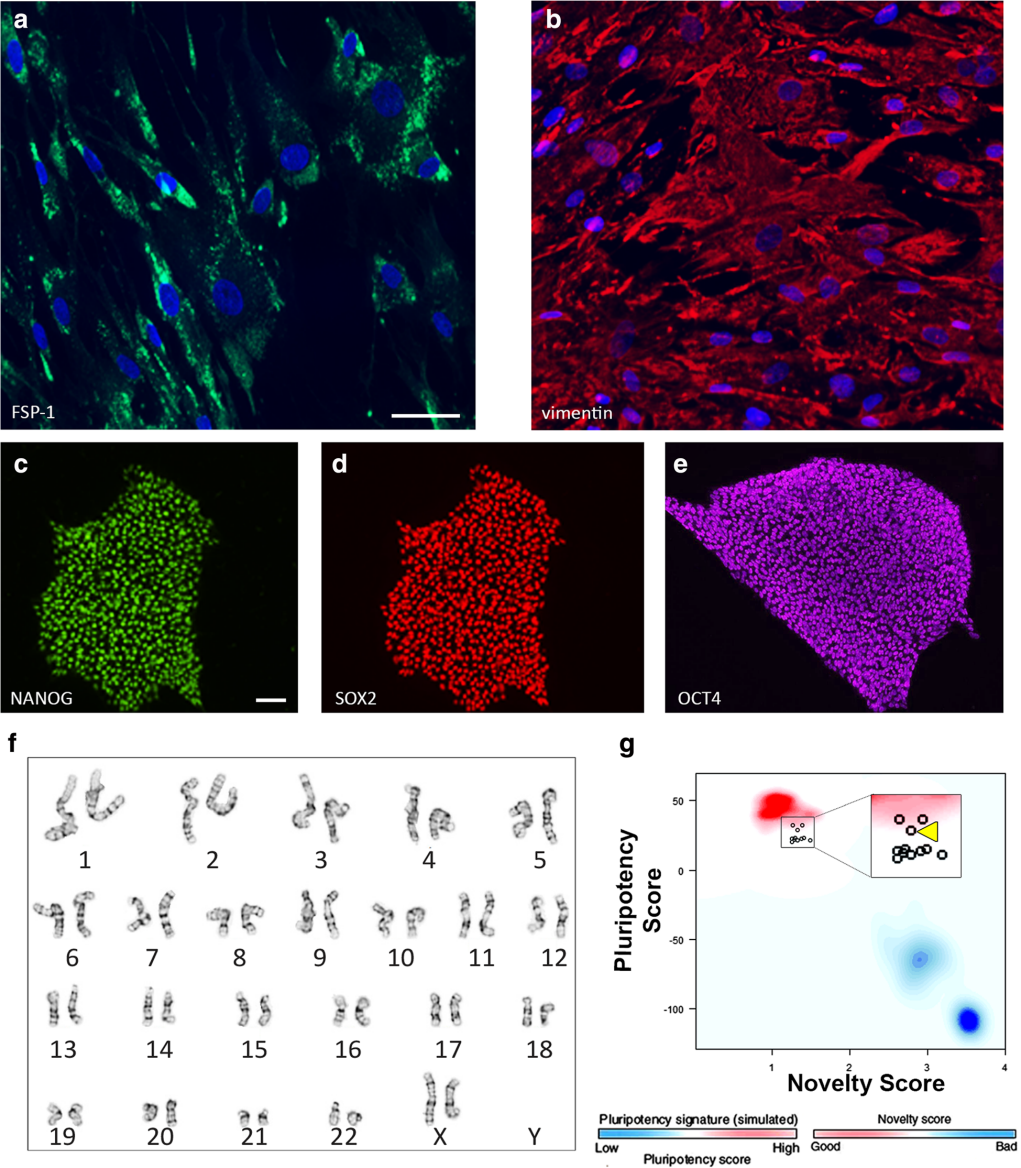
Reprogramming of human fibroblasts into induced pluripotent stem cells. **a**, **b** Fibroblast cells immunolabeled with antibodies against FSP-1 (**a**, green) or vimentin (**b**, red). DAPI (blue) was used to counterstain the nuclei. **c**–**e** iPSCs immunolabeled with antibodies against NANOG (**c**), SOX2 (**d**), and OCT4 (**e**). Expression of these proteins indicates the identity of iPSCs derived from fibroblast reprogramming. **f** Normal karyotype of an iPSC line determined by G-banding. **g** Results from PluriTest assay show that our iPSCs have a pluripotent signature (high pluripotency score and low novelty score), and cluster with well-characterized bona-fide iPSC and ESC lines (red background hint; iPSC line 2 is indicated by a yellow arrowhead in the magnified panel) and not with partially differentiated pluripotent cells or somatic tissue (blue background hint). Scale bars: **a**, **b**, 50 μm; **c**–**e**, 20 μm. FSP-1 fibroblast-specific protein-1

Mechanical (i.e., scalpel) and enzymatic (i.e., collagenase) methods were used to isolate fibroblasts from human dermal tissue obtained via skin biopsy. The identity of fibroblasts was confirmed by immunolabeling for FSP-1 (Fig. 1a) and for vimentin (Fig. 1b). Immunolabeling revealed that ≥ 95% of the cells expressed both FSP-1 and vimentin. The fibroblasts could be cultured and propagated using xeno-free conditions; they remained stable in culture for at least five passages. To generate iPSCs, using a nonintegrating method, we transfected the fibroblasts with the VEE RNA vector [27], which induces exogenous expression of pluripotency markers, including OCT4, KLF4, SOX2, and GLIS1. Following puromycin selection, cells were replated and cultured on Matrigel for 3–4 weeks, when the formation of iPSC colonies was evident. The identity of iPSCs was confirmed by immunolabeling for pluripotency markers, including NANOG, SOX2, and OCT4 (Fig. 1c–e). Normal karyotype was shown by G-banding (Fig. 1f). Additionally, the iPSCs were capable of generating cells from all three embryonic germ layers in a bioinformatics assay for pluripotency, PluriTest (Fig. 1g).

### Differentiation and characterization of iPSC-RPE cells

**Fig. 2:**
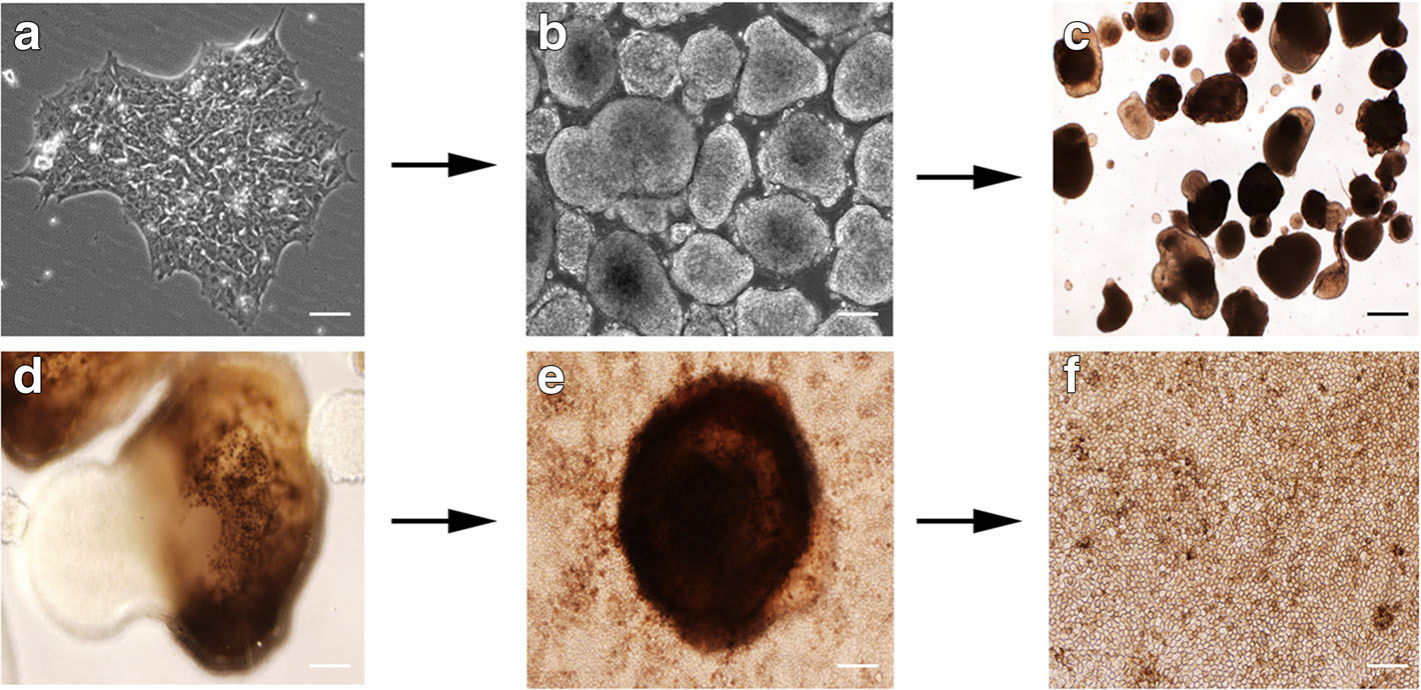
Differentiation of iPSCs into RPE cells. The differentiation process begins with colonies of iPSCs plated initially on Matrigel (**a**) and subsequently detached to be cultured as EBs in suspension, and in the presence of NIC for 2 weeks (**b**). **c**, **d** The EBs became pigmented following a 2-week exposure to Activin A, TGFB1, and FGF2. The pigmented regions in the EBs were mechanically dissected and plated in RPE medium (**e**) giving rise to a monolayer of pigmented cells with a cobblestone appearance that can be further purified and expanded (**f**). Scale bars: **a**, **b**, 20 μm; **c**, 50 μm; **d**, 10 μm; **e**, 100 μm; **f**, 50 μm

**Fig. 3:**
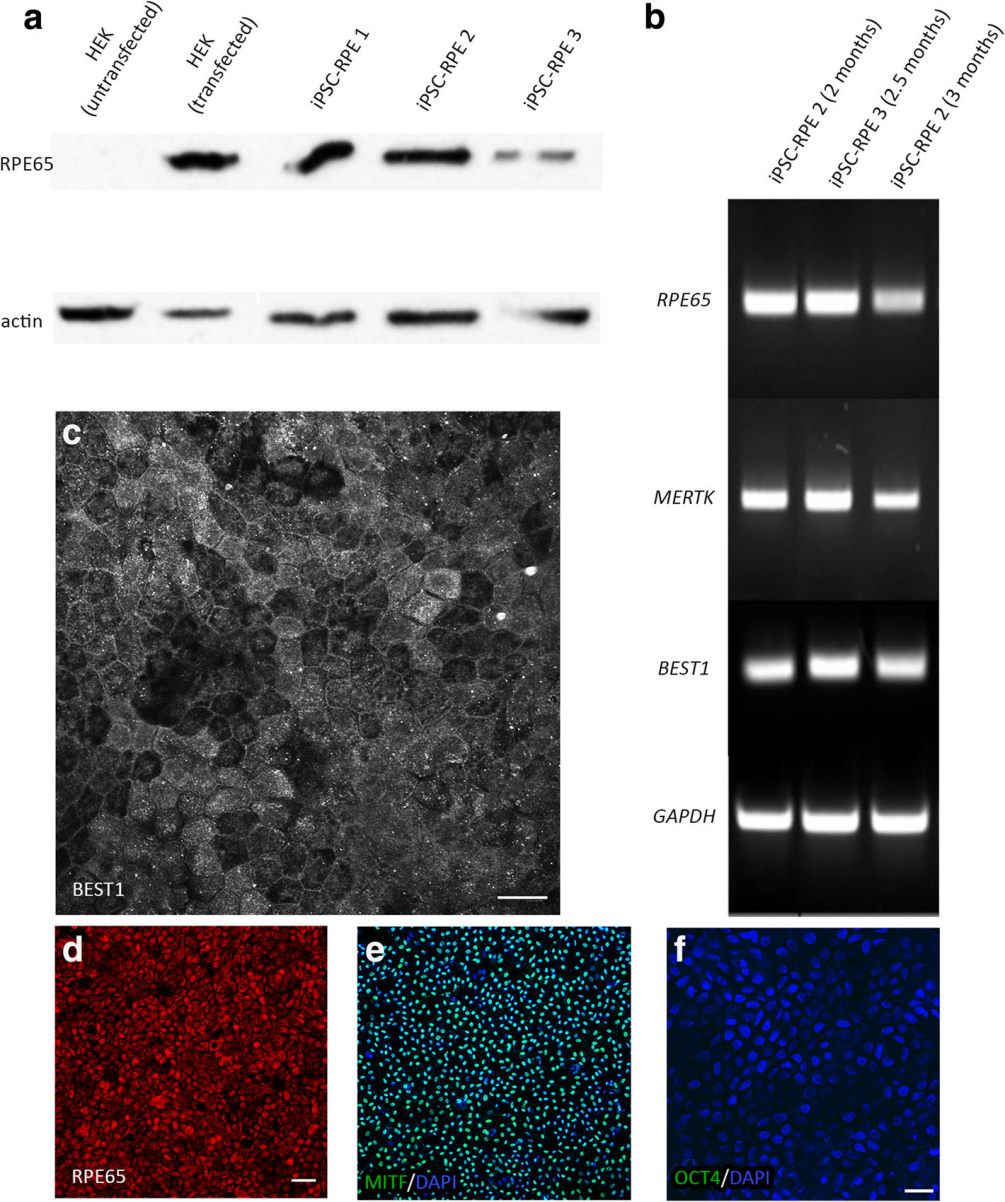
Specific protein and gene expression profiling of iPSC-derived RPE cells. **a** Expression of RPE65 by 2.5–3-month cultures of RPE cells from three different iPSC lines, indicated by an immunolabeled western blot image. Actin was used as a loading control. **b** Expression of *RPE65*, *MERTK*, and *BEST1* genes in iPSC-RPE cells cultured for 2–3 months, detected by RT-PCR. GAPDH was used as a loading control. **c**–**e** Expression of the RPE-specific proteins BEST1 (**c**), RPE65 (**d**), and MITF (**e**), indicated by immunofluorescence. **f** iPSC-RPE cells showed no expression of the pluripotency marker OCT4; nuclei labeled with DAPI. Scale bars: **c**, 20 μm; **d**, **e**, 50 μm; **f**, 20 μm. HEK human embryonic kidney cells, iPSC induced pluripotent stem cell, RPE retinal pigment epithelium, MERTK MER proto-oncogene, tyrosine kinase, BEST1 bestrophin 1, GAPDH glyceraldehyde-3-phosphate dehydrogenase, MITF microphthalmia-associated transcription factor

To derive RPE cells, colonies of iPSCs (Fig. 2a) were transferred as suspension cultures in low-adherent dishes to generate EBs (Fig. 2b). After 2 weeks in culture, Activin A, TGFB1, and FGF2 were added to the medium for a period of 2 weeks. Following the removal of these growth factors, we observed the appearance of pigmented EBs in as early as 4 weeks (Fig. 2c). The pigmented regions (Fig. 2d) of the EBs were dissected and plated as adherent cultures in RPE medium. The pigmented regions adhered as a large mass from which pigmented cells began to proliferate and migrate near the periphery (Fig. 2e). The pigmented cells were enzymatically passaged and cultured until confluent monolayers of pigmented cells with cobblestone morphology appeared (Fig. 2f; Additional file 1A, B). Pigmented cells were derived from three individual iPSC lines, which, by western blotting analysis, were confirmed to express the RPE-specific protein, RPE65 (Fig. 3a). Additionally, after 2–3 months in culture, RPE cells from multiple iPSC lines showed robust expression of RPE genes, including *RPE65*, *MERTK*, and *BEST1*, by semi-quantitative RT-PCR (Fig. 3b). By immunocytochemistry, these pigmented cells showed expression of RPE signature proteins, such as BEST1, RPE65, and MITF (Fig. 3c–e), and no detectable expression of the pluripotency marker OCT4 (Fig. 3f). Taken together, these results indicate that the pigmented cuboidal cells derived from our integration-free iPSCs have expression characteristics of RPE cells.

### Phagocytosis of photoreceptor outer segments by iPSC-RPE cells

**Fig. 4:**
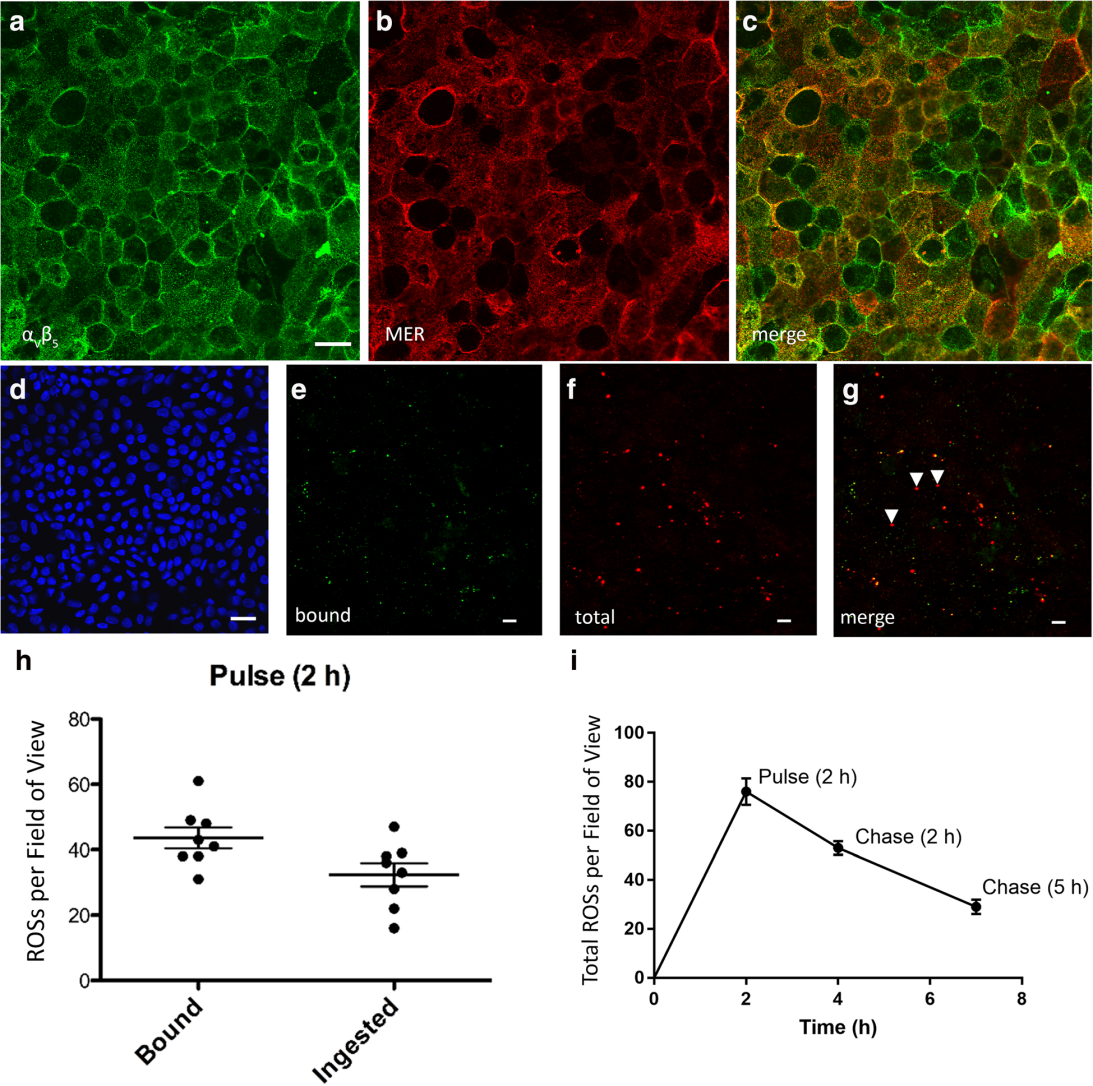
Phagocytosis of photoreceptor outer segments by iPSC-RPE cells in vitro. Immunofluorescence labeling of iPSC-RPE cells for integrin α_v_β_5_ (**a**) and MERTK (**b**). These receptors are responsible for the binding and internalization of POSs, respectively, and are present on the apical surface. **c** Image merge of **a** and **b**. The ability of iPSC-RPE cells to phagocytize POSs was tested in vitro by challenging the cells with POSs isolated from porcine retinas. **d** Micrograph of iPSC-RPE cells that have not been exposed to POSs and labeled with RHO antibody. Nuclei counterstained with DAPI. **e** Representative image of bound ROSs, labeled with an antibody against RHO and a green secondary antibody, prior *to* cell permeabilization. **f** Representative image of all (internalized and bound) ROSs labeled with the same RHO antibody, but with a red secondary antibody, following cell permeabilization. **g** When merged, the surface-bound ROSs appear yellow while internalized ROSs appear red. A few internalized ROSs are indicated by white arrowheads. **h** Quantification of bound and ingested ROSs was performed using the ImageJ software to count RHO-positive particles with diameters greater than 0.5 μm. **i** Total ROSs per field of view were quantified after the 2-h pulse, and after a 2-h and 5-h chase. Phagosome counts were obtained from six to eight individual fields of view, with each field containing ≥ 100 cells. **h**, **i** Data represent mean ± SD. Scale bars: **a**, **d**, 20 μm; **e**–**g**, 5 μm. ROS rod outer segment

One of the primary functions of the RPE is the phagocytosis of distal tips of POS disk membranes [28]. Defects in this process have been shown to cause photoreceptor degeneration [29], with defects in the degradation phase linked to RPE pathogenesis [18, 30]. The initial process of phagocytosis by the RPE involves binding followed by ingestion of POS membranes, two events in which integrin α_V_β_5_ and MERTK receptors have been shown to participate, respectively [31, 32]. While RPE cells derived from stem cells have been shown to phagocytose POS membranes previously [33], here we have explored this function more extensively. We first tested whether our iPSC-RPE cells expressed these receptors at their surface. Immunocytochemistry confirmed the expression of integrin α_V_β_5_ (Fig. 4a) and MERTK (Fig. 4b), which colocalized at the surface of the iPSC-RPE cells (Fig. 4c). To test the phagocytic function in our iPSC-RPE cells, we cultured the cells on laminin-coated Transwell inserts, and challenged them with porcine POSs for 2 h to allow sufficient time for binding and ingestion to occur. Following the challenge, and after extensive washes to remove unbound POSs, some cultures were allowed additional time (2 or 5 h) to degrade the POSs they had ingested. Using a double immunolabeling strategy for RHO, a protein abundant in the ROSs but not expressed by the RPE (Fig. 4d), we first labeled ROSs bound to the surface of the RPE cells (Fig. 4e), and then, after permeabilization of the cells, labeled all ROSs (bound and ingested) (Fig. 4f). Thus, we were able to discriminate between bound and ingested ROSs (Fig. 4g). We found that when the iPSC-RPE cells were cultured on laminin-coated Transwell inserts (to achieve a polarized monolayer of cells), approximately 60% of the ROSs were bound to the surface of the cells while approximately 40% had been internalized at the end of a 2-h challenge (Fig. 4h). When given additional time (chase) after the POS challenge, the iPSC-RPE cells were able to degrade more than 50% of the total ROS phagosomes during a 5-h chase period (Fig. 4i, Additional file 2). These results demonstrate that GMP-compatible iPSC-RPE cells are capable of binding, internalizing, and subsequently degrading ROSs. The rate of ROS phagosome degradation was much faster than that reported for human RPE cells from immortalized cell lines; no significant degradation of opsin was detected in human d407 cells until more than 8 h after the removal of unbound POSs [34].

### Organelle trafficking in live iPSC-RPE cells

**Fig. 5:**
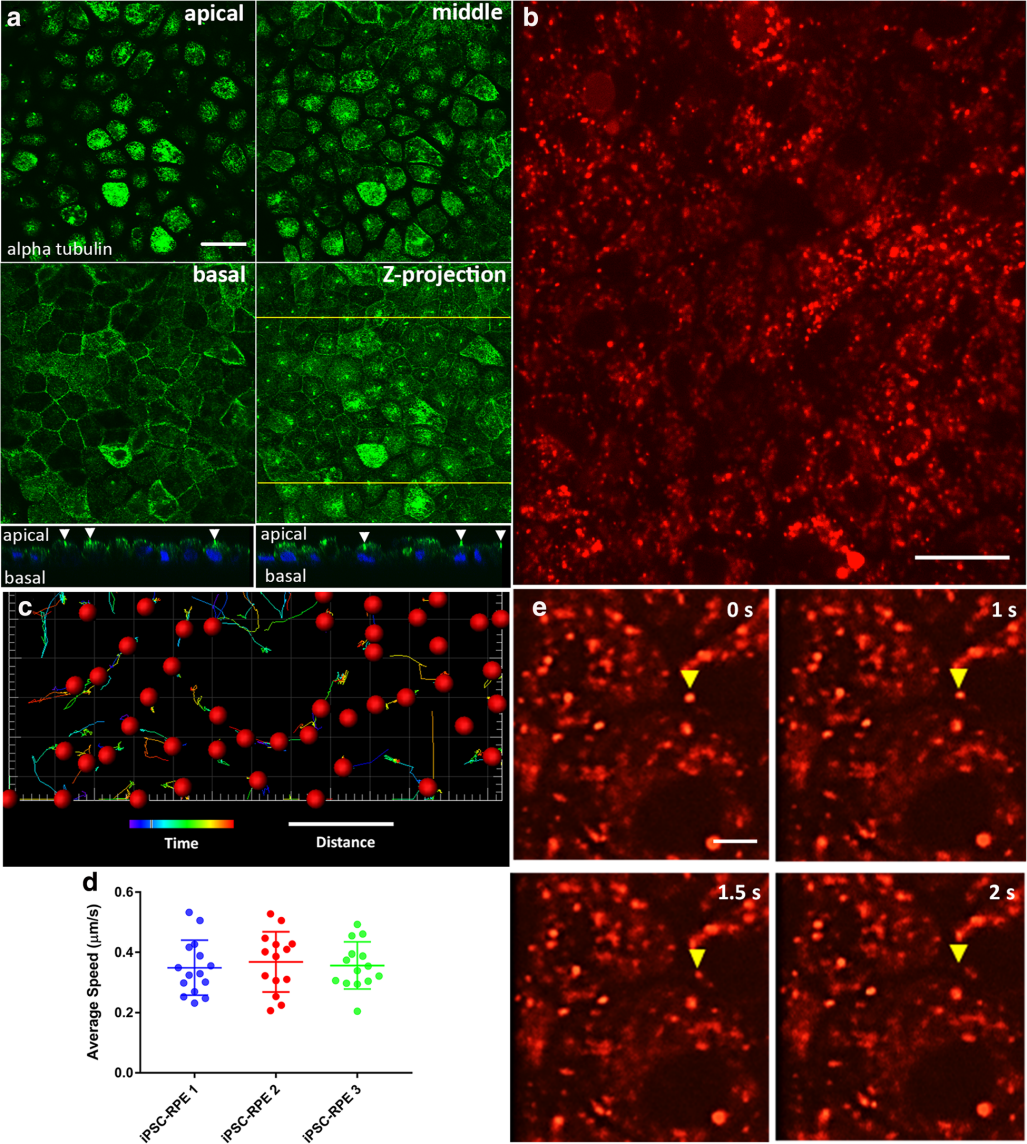
Microtubule organization and trafficking of endolysosomes in iPSC-RPE cells. **a** Microtubule organization in the iPSC-RPE cell bodies illustrated by immunostaining of alpha tubulin. Single-plane confocal microscopy images (2-μm apart) represent the apical and basal regions of the cell bodies, plus one plane in between (middle). The apical region of the cells is dominated by horizontal microtubules while the basal region is dominated by vertical microtubules. A *z* projection of the three panels is shown in the fourth panel. Below are images in two *z* planes at the yellow lines in the *z*-projection image, showing primary cilia (indicated by white arrowheads) emanating from the apical surface of the RPE cells. **b** Still image from a movie of iPSC-RPE cells that were incubated with red LysoTracker to label endolysosomes (see Additional file 4 for a similar movie). **c** Trajectory and movement analysis of a population of endolysosomes, using a spots and tracks analysis (Imaris), following movie acquisition over a 25-s interval. The tracks represent the trajectories of the organelles, while their colors are indicative of how far (in terms of time) they are with respect to the 25-s movie, with cool colors being closer to the beginning of the movie, and hot colors being closer to the end of the movie. **d** Time-lapse images from a movie showing vertical movement of a labeled organelle (yellow arrowhead). Each panel represents the same *z* plane at different times. The organelle moves out of the plane after 2 s, indicating that it is traversing different *z* planes. Scale bars: **a**, **b**, 20 μm; **c**, 25 s (time), 5 μm (distance); **e**, 5 μm. iPSC induced pluripotent stem cell, RPE retinal pigment epithelium

Normal trafficking of organelles is essential for the function of RPE cells [35], and is a sensitive measure of the polarized organization of their cytoskeleton, as well as their metabolic health. In particular, the process of POS phagocytosis relies on the trafficking of POS-derived phagosomes, and their interaction with degradative organelles such as endolysosomes for POS clearance [36, 37]. When this trafficking is perturbed, RPE health becomes compromised, with the development of AMD-like pathogenesis [18, 38]. Prior to examining organelle trafficking in our iPSC-RPE cultures, we used immunofluorescence confocal microscopy to evaluate the organization of the microtubules. This organization is a sensitive indicator of epithelial cell polarization. In addition, the microtubules serve as essential cytoskeletal elements for trafficking POS phagosomes and endolysosomes in the RPE [18, 39]. Transverse sections of alpha tubulin immunolabeling, at different depths in the cell body (Fig. 5a; Additional file 3), show microtubules that in the apical region extended into the plane of the section, indicating a horizontal orientation, and in the basal region appeared more punctate, indicating a vertical orientation, most concentrated adjacent to the plasma membrane. Additionally, the staining revealed primary cilia, indicated by white arrowheads in *z*-plane views under Fig. 5a, emanating from the apical surface of the iPSC-RPE cells. This organization of microtubules is consistent with that observed in other polarized epithelial cells [40–43]. To evaluate the trafficking of organelles in our iPSC-RPE cultures, we incubated the cells with LysoTracker, which labels acidic organelles such as endosomes and lysosomes, and used high-speed spinning disk confocal microscopy to perform live-cell imaging (Fig. 5b; Additional file 4). Following movie acquisition, the tracks of labeled organelles were analyzed during a time interval of 20–40 s (Fig. 5c). We found that the average speed of labeled organelles ranged from 0.2 to 0.5 μm/s, and this range was similar among RPE cells derived from the three independent iPSC lines (Fig. 5d). Similar speeds have been observed for LAMP2-positive organelles in polarized primary cultures of porcine RPE [44], and they represent a range consistent with microtubule transport. Interestingly, many of these labeled organelles can be seen moving in and out of the *x*–*y* plane, suggesting vertical motility along the *z* axis (Fig. 5e), consistent with the presence of vertical microtubules, which are a hallmark of well-polarized epithelial cells.

### Transepithelial resistance

**Fig. 6:**
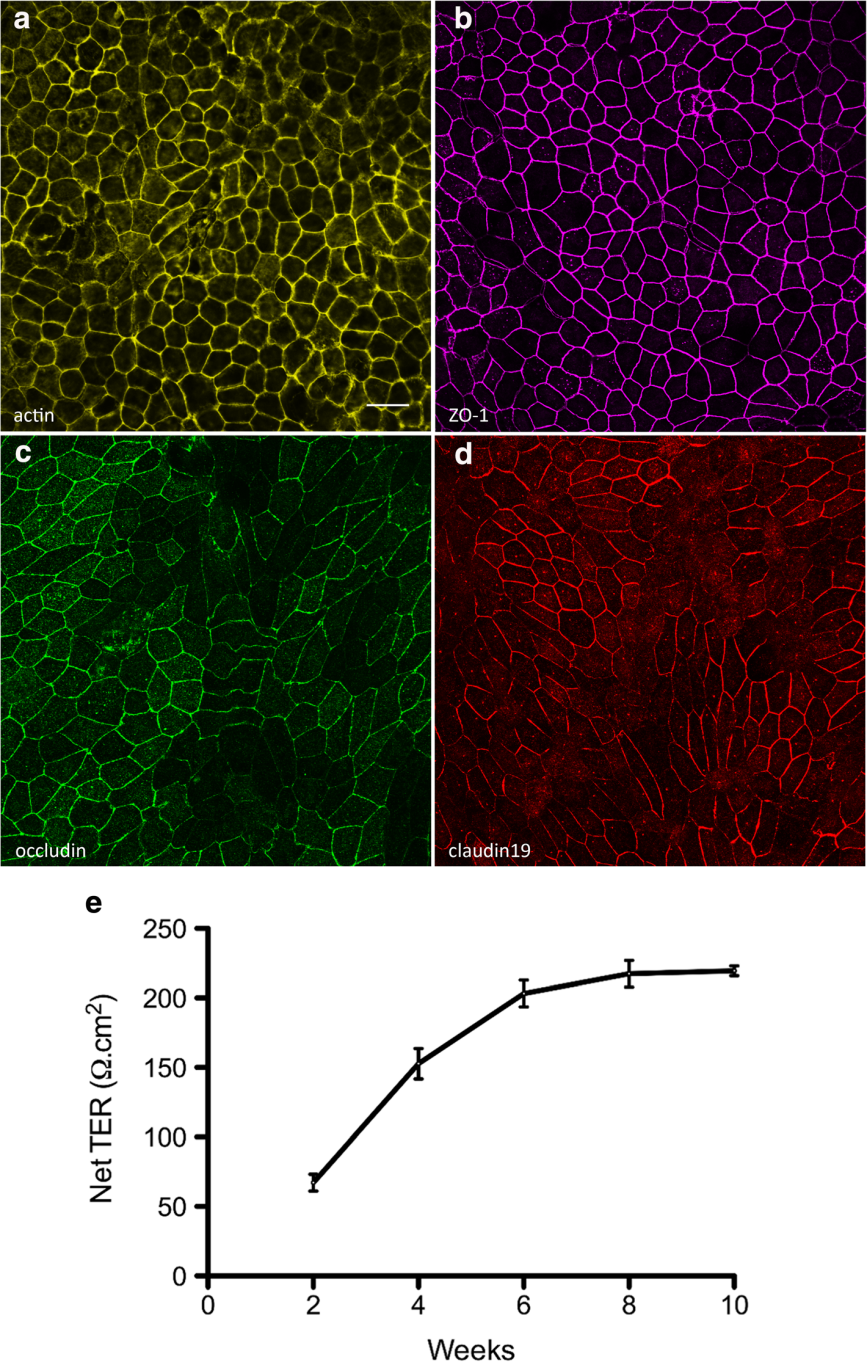
Tight junction proteins and the transepithelial resistance of iPSC-RPE. RPE cells were cultured on laminin-coated Transwell inserts. **a** Phalloidin labeling demonstrates the cortical arrangement of actin filaments in iPSC-RPE. **b**–**d** Immunostaining of the iPSC-RPE cells revealed surface expression of the tight junction proteins ZO-1 (**b**), occludin (**c**), and claudin19 (**d**). **e** To assess the barrier function of the iPSC-RPE cells, the resistance across monolayers of cells cultured on laminin-coated Transwell inserts was measured at 2-week intervals, following initiation of the cultures. The net TER was determined by subtracting the resistance across a laminin-coated Transwell insert lacking cells, and multiplying by the surface area of the insert (0.33 cm^2^). The net TER of the iPSC-RPE cells steadily increased between weeks 2 and 8, until it reached a maximal value of just above 200 Ω.cm^2^. Resistance recordings were made from four individual cultures. **e** Error bars represent the mean ± the SEM. Scale bars: **a**–**d** = 20 μm. ZO-1 zonula occludens-1, TER transepithelial resistance

The RPE is situated between the photoreceptors and the choriocapillaris, thereby serving as a blood–retinal barrier [45]. To perform such a function, the RPE forms tight junctions near its apical surface to tightly regulate the flux of ions, molecules, and fluid between the inner retina and the blood supply [46]. To test whether our iPSC-RPE cells expressed the proteins necessary to form these tight junctions, we cultured the cells on laminin-coated Transwell inserts and performed immunocytochemistry to examine the expression of tight junction proteins, including ZO-1, occludin, and claudin19. The immunostaining revealed that the cells, which have proper cortical arrangement of actin filaments (Fig. 6a; Additional file 1C, D), expressed the three tight junction proteins, with normal localization at the plasma membrane (Fig. 6b–d; Additional file 1E–H).

The resistance across the RPE layer provides a measure of the function of the tight junctions. To measure the TER, we cultured our iPSC-RPE cells on laminin-coated Transwell inserts, and made recordings at 2-week intervals. We observed a steady increase in the TER until it reached a maximal level of slightly above 200 Ω.cm^2^, by 8 weeks of culture (Fig. 6e). This value is consistent with the reported net TER of human RPE in vivo, which ranges from 150 to 200 Ω.cm^2^ [47–49]. Collectively, the expression of tight junction proteins and the measured TER indicate that the iPSC-RPE cells are capable of establishing an appropriate epithelial barrier in vitro.

### Integration of human iPSC-RPE into the murine RPE in vivo

**Fig. 7:**
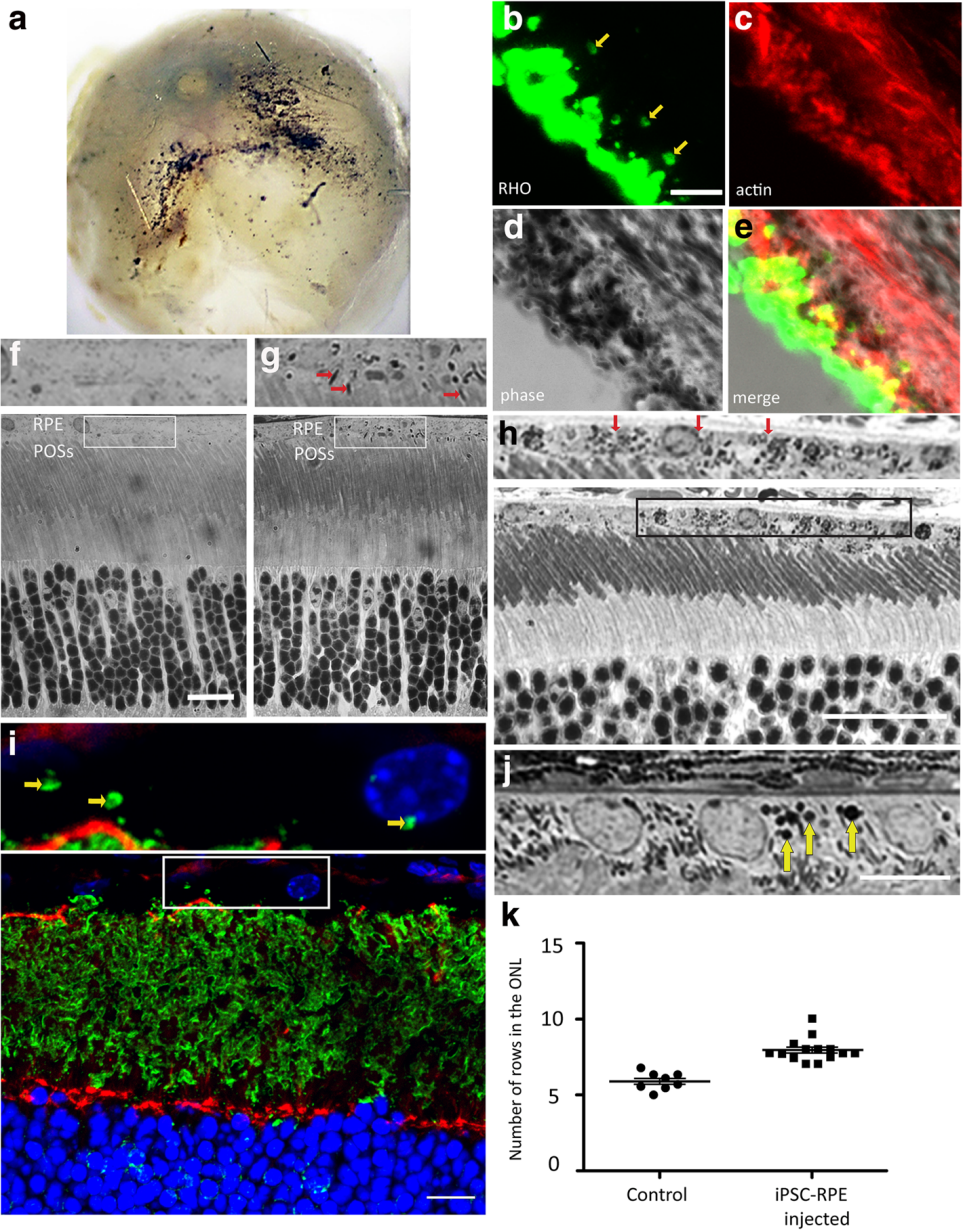
Transplantation of human iPSC-RPE cells into murine eyes. **a**–**h** Injection of pigmented human iPSC-RPE cells into the subretinal space of albino mouse eyes. In the examples illustrated, injections were performed at P14. **a** Posterior segment of a whole eye, 28 days after injection, showing evidence of pigment. **b**–**d** Thick retinal cryosection, 28 days after injection, labeled with anti-RHO (**b**) and phalloidin (**c**), shown under phase contrast (**d**). **e** Merge of RHO and actin labeling, together with phase contrast imaging. RHO labels ROSs, as well as phagosomes in the RPE (yellow arrows in **b**; inner retina is lower left). Phalloidin labeling of f-actin delineates the apical RPE. Pigmentation in the RPE cells is evident by the phase-contrast imaging. Pigmented RPE cells are therefore evident in the RPE layer, adjacent to the labeled ROSs, and contain ROS phagosomes. **f**–**h** Retinal semi-thin sections from a P40 noninjected albino mouse (control) (**f**), and from albino mice fixed 28 days (**g**) or 205 days (**h**) after injection. Boxed regions in the lower panels are shown at higher magnification in the upper panels. iPSC-RPE cells are identifiable by the presence of melanosomes in the apical processes (red arrows in **g**), adjacent to the tips of the POSs, in addition to the cell body. Pigmented iPSC-RPE cells (red arrows in **h**) remain integrated in the retina, 200 days post injection. **i**–**k** Injection of pigmented human iPSC-RPE cells into the subretinal space of *Mertk* ^–/–^ mouse eyes. In the examples illustrated, injections were performed at P10, and retinas were examined 27 days after injection, at P37. **i** Fluorescence micrograph of retinal section labeled with anti-RHO (green) and phalloidin (red). Nuclei were counterstained with DAPI. Yellow arrows in an enlarged region (upper) indicate RHO-positive phagosomes in transplanted iPSC-RPE cells. **j** Micrograph of toluidine blue-stained semi-thin section (yellow arrows indicate ROS phagosomes). **k** Quantification of photoreceptor nuclei in semi-thin sections of the central retina. For the data illustrated, injections were made with PBS (control) or iPSC-RPE cells (*p* = 0.0001) at P10, and sections were obtained 27 days later, at P37. For reference, the central region of a 1–2-month-old WT retina contains ~ 10 rows of photoreceptor nuclei (e.g., **f**). Scale bars: **b**–**e**, 5 μm; **f**–**h**, 20 μm; **i**, **j**, 10 μm. iPSC induced pluripotent stem cell, ONL outer nuclear layer, POS photoreceptor (rod and cone) outer segment, RHO rhodopsin, RPE retinal pigment epithelium

Transplantation of healthy and functional RPE cells has the potential of treating an eye with diseased or dysfunctional RPE. To test for integration of iPSC-RPE cells into an RPE layer in vivo, we injected suspensions of RPE cells into the subretinal space of albino mouse eyes (Fig. 7a). After various intervals, retinal cryosections were obtained and fluorescently labeled with RHO antibodies and phalloidin for f-actin. Images of phase contrast merged with RHO and actin fluorescence revealed that the injected pigmented iPSC-RPE cells, delineated by the phalloidin labeling for actin, had integrated into the host retina, and were situated apical to the outer segments of the photoreceptors, marked by the RHO labeling (Fig. 7b–e). Additionally, the pigmented iPSC-RPE cells also contained phagosomes, derived from ingested ROSs, suggesting that the integrated cells were capable of performing phagocytosis (Fig. 7b).

Light microscopy on semi-thin sections from injected eyes confirmed the presence of melanosome-containing cells that were absent in noninjected eyes (Fig. 7f, g). Following the longest interval post injection that we tested, 205 days, these pigmented cells remained detectable in the retinas of injected albino eyes (Fig. 7h). It is important to note that the melanosomes were evident in the apical processes of the RPE cells, in addition to the cell body (Fig. 7g, red arrows). Localization to the apical processes, and with an orientation that is approximately parallel to that of the POSs, has been shown to require the molecular motor, myosin-7a, in mouse [50, 51] and human [52] RPE, and demonstrates that the melanosomes are endogenous to the RPE cells. An alternative explanation for the presence of melanosomes in the RPE is that albino host cells ingested debris from the injected pigmented cells. While such ingestion can happen, it results in the retention of the melanosomes only within vacuoles in the RPE [26]. These results confirm that a suspension of iPSC-RPE cells can be targeted to the outer retina, where the cells are capable of integrating into the host tissue, and can remain stable for a significant period of time after the injection procedure.

### In-vivo rescue of retinal degeneration by iPSC-RPE cells

RPE dysfunction or pathology has been indicated in numerous forms of retinal degeneration, including retinitis pigmentosa, Best disease, Stargardt’s disease, and AMD. It is therefore appealing to use iPSC-derived RPE for autologous transplantations to rescue RPE functions lost in some of these degenerative diseases. In our in-vivo studies, we used the *Mertk* ^–/–^ mouse as a model of retinal degeneration, with RPE dysfunction, and tested whether our iPSC-RPE cells could rescue the inherent phagocytosis deficiency of the RPE.

We injected suspensions of 50,000 iPSC-RPE cells into the subretinal space of *Mertk* ^–/–^ mice at age P10, long before retinal degeneration has been observed to occur in this model. The animals were sacrificed 27 days post injection, and thick cryosections and semi-thin Epon sections were obtained from the retinas. The cryosections were stained with phalloidin to identify the apical region of the RPE, and an antibody against RHO to identify phagosomes of ROSs phagocytized by the RPE. In the semi-thin sections, phagosomes were identified by heavy staining with toluidine blue. MERTK functions in the ingestion of POSs by the RPE, so that the *Mertk* ^–/–^ RPE lacks phagosomes [53], and the presence of numerous phagosomes in the RPE layer is an indicator of transplanted functional iPSC-RPE. Figure 7i shows phagosomes that have been identified by RHO immunolabeling. Figure 7j shows a cluster of toluidine blue-stained phagosomes (yellow arrows) in a semi-thin section of the RPE layer. The semi-thin sections were also used to quantify the number of rows of photoreceptor nuclei in the outer nuclear layer (ONL) of the central retina, near the injection site. This quantification showed that *Mertk* ^–/–^ mice injected with iPSC-RPE cells had more rows of nuclei in the ONL, relative to noninjected mice (Fig. 7k). Overall, these results demonstrate the ability of iPSC-RPE to rescue a lost function of the RPE, in vivo, and partial rescue of photoreceptor degeneration, in a mouse model of inherited retinal degeneration.

## Discussion

Numerous in-vitro cell models have been used to study basic human RPE cell biology, including primary cultures from donor tissues, and immortalized cell lines, such as ARPE-19, d407, and hTERT-RPE1 [54, 55]. Although RPE cultures from human fetal tissue do mimic in-vivo characteristics well [56, 57], their supply is limited, and a large supply of isogenic cell cultures is not feasible. On the other hand, RPE cultures from immortalized cell lines have been reported to fall short of in-vivo characteristics, including signature gene expression, robust TER, structural polarity, and functional aspects such as kinetics of POS phagosome degradation [34, 58–60]. Many of these limitations have been mitigated by using human pluripotent stem cells to obtain RPE cells in large quantities for cell culture studies as well as therapeutic transplantation. Here, we have advanced the use of iPSC-RPE cells by a GMP-compatible method of iPSC generation, coupled with novel analyses of critical cell biological functions.

RPE cells were one of the first cell types to be isolated from pluripotent stem cells, due to their readily discernible pigmentation [12], and a variety of protocols have been developed to improve and hasten this process [14–17]. Some of these protocols have generated RPE cells from integration-free iPSCs [61, 62] as these cells are more likely to be free of mutations due to the reprogramming process, and are therefore better candidates for transplantation purposes. Here, we have used GMP-compatible conditions to differentiate RPE from iPSCs that have been generated using integration-free reprogramming, and demonstrated that the iPSC-RPE cells possessed key characteristics that will likely be essential to their function in clinical uses.

Cultures of iPSC-RPE cells have been characterized with respect to gene expression, the presence of selected protein markers, and some functional assays [25, 63]. An extensive recent study focused on ATP-dependent RPE physiology [64]. The particular focus in the present study has been on aspects of RPE cell biology that are critical for retinal health. By week 7 of the differentiation process, we observed robust expression of signature RPE proteins, including BEST1, RPE65, and MITF. At that time, the cytoskeleton of the cells resembled that of an epithelium, with actin filaments organized at the cortex, and microtubules arranged horizontally in the apical cell body and vertically throughout the cell body [40–43]. The epithelial arrangement of the cytoskeleton was underscored by our live-cell imaging analysis, in which 8-week cultures of iPSC-RPE cells exhibited lateral and vertical motility of endolysosomes. The observed intracellular motility is a critical indicator of RPE cell health. Each RPE cell in the human retina must efficiently degrade phagosomes derived from 30 POSs on a daily basis [65]. Defects in motor proteins that drive organelle motility in the RPE have been shown to compromise phagosome degradation, and lead to retinal pathology, including symptoms of AMD, which is potentially the most significant target disease of RPE transplantation [18, 38].

In addition, the iPSC-RPE cells showed both normal expression and localization of tight junction proteins, including ZO-1, occludin, and claudin19, by week 7 of the differentiation [66, 67]. This was reflected functionally by the TER of the cultures, which by week 7 had reached 200 Ω.cm^2^. Finally, by week 8 of the differentiation process, we observed surface localization of the receptors integrin α_v_β_5_ and MERTK, which participate in the binding and ingestion of POSs, respectively [31, 32]. These receptors were shown to be functional in assays that demonstrated the phagocytosis of POSs by iPSC-RPE cells, with kinetics comparable to that in vivo.

These results support the use of iPSC-RPE cells for in vitro studies of pathogenicity in RPE disease. In addition, a clinically relevant goal for RPE cells derived from iPSCs is to be able to transplant these cells into patients with maculopathies, where RPE dysfunction or dystrophy contributes to the overall pathology. The RPE can be transplanted as either an intact sheet of cells or as a suspension of dissociated cells [68, 69]. A major concern with the suspension method is that the cells may not integrate properly in order to perform their function. Here, we demonstrated that a suspension of iPSC-RPE cells can integrate into the host RPE monolayer, and is capable of partially rescuing a critical function of the RPE that has been compromised due to a genetic defect. The ability of iPSC-RPE cells to rescue the pathology associated with certain maculopathies is likely to be dependent on the quality of the RPE cells that are used. In this study, we placed a special emphasis on characterizing the highly sensitive cell biological characteristics of iPSC-RPE cells, such as intracellular trafficking, to obtain well differentiated cultures suitable for transplantation.

## Conclusions

We have generated lines of iPSC-RPE cells, using a nonintegrating method of cellular reprogramming and a differentiation protocol, under GMP-compatible conditions. The iPSC-RPE cells were shown to possess important RPE characteristics of normal RPE, including, for the first time, critical aspects of RPE cell biology. Thus, this report documents a significant addition to a growing body of literature, validating the differentiation of bona-fide RPE cells from stem cells, for both disease-in-a-dish modeling and therapeutic transplantation.

## Additional files

Additional file 1: Is a figure showing (**A**, **B**) brightfield micrographs of iPSC-RPE 1 (**A**) and iPSC-RPE 3 (**B**), illustrating the pigmentation and cobblestone morphology of the cells; (**C**, **D**) phalloidin labeling of iPSC-RPE 1 (**C**) and iPSC-RPE 3 (**D**), illustrating the cortical arrangement of actin filaments in the cells; (**E**–**H**) Immunofluorescence micrographs, illustrating expression of the tight junction proteins, ZO-1 (**E**, **F**) and occludin (**G**, **H**), in iPSC-RPE 1 and 3. Scale bars: **A**, **B**, 60 μm; **C**–**H**, 20 μm. (TIF 5893 kb)

Additional file 2: Is a figure showing quantification of phagocytosis in iPSC-RPE 1 and 3 exposed to porcine POSs for 2 h (pulse), washed extensively to remove unbound POSs, and then allowed either a 2-h or 5-h chase period to ingest and degrade the POSs. Graph shows the total number of ROSs quantified from confluent fields of view after the pulse and the two separate chase periods. Data represent mean ± SD. (TIF 89 kb)

Additional file 3: Is a figure showing alpha tubulin labeling in iPSC-RPE 1 (**A**, **C**, **E**, **G**) and iPSC-RPE 3 (**B**, **D**, **F**, **H**) showing the arrangement of microtubules in an apical region (**A**, **B**), middle region (**C**, **D**), and basal region (**E**, **F**) of the cells. The apical region is dominated by horizontally-oriented microtubules whereas the basal region consists mainly of vertically-oriented microtubules. (**G**, **H**) *z* projections; *z* planes at the locations of the yellow lines illustrating the presence of primary cilia (indicated by white arrowheads) on the apical surface of the iPSC-RPE cells. Scale bars: 20 μm. (TIF 4278 kb)

Additional file 4: Is a movie showing live-cell imaging of endolysosomes, labeled with LysoTracker, showing the 4D movement of these organelles in iPSC-RPE cells cultured on laminin-coated chambered coverglass. The movie was acquired at 1.9 frames per second using a spinning disk confocal microscope, and plays at 10 frames per second. Scale bar, 5 μm. (MP4 727 kb)

## Abbreviations

AMD: Age-related macular degeneration
BEST1: Bestrophin 1
BSA: Bovine serum albumin
COSs: Cone outer segments
DAPI: 4, 6′-Diamino-2-phenylindole
DMEM/F12: Dulbecco’s Modified Eagle Medium: Nutrient Mixture F-12
EB: Embryoid body
ESC: embryonic stem cell
FBS: Fetal bovine serum
FGF2: Fibroblast growth factor 2
FSP-1: Fibroblast-specific protein 1
GMP: Good Manufacturing Practice
iPSC: Induced pluripotent stem cell
MERTK: MER proto-oncogene, tyrosine kinase
MITF: Microphthalmia-associated transcription factor
NEAA: Nonessential amino acids
NGS: Normal goat serum
ONL: Outer nuclear layer
PBS: Phosphate-buffered saline
POS: Photoreceptor (rod and cone) outer segment
RHO: Rhodopsin
ROS: Rod outer segment
RPE: Retinal pigment epithelium
RT-PCR: Reverse transcription polymerase chain reaction
TER: Transepithelial resistance
TGFB1: Transforming growth factor beta 1
ZO-1: Zona occludens-1
