# Correction to: Differentiation of RPE cells from integration-free iPS cells and their cell biological characterization

**DOI:** 10.1186/s13287-019-1147-7

**Published:** 2019-02-12

**Authors:** Roni A. Hazim, Saravanan Karumbayaram, Mei Jiang, Anupama Dimashkie, Vanda S. Lopes, Douran Li, Barry L. Burgess, Preethi Vijayaraj, Jackelyn A. Alva-Ornelas, Jerome A. Zack, Donald B. Kohn, Brigitte N. Gomperts, April D. Pyle, William E. Lowry, David S. Williams

**Affiliations:** 1 Stein Eye Institute and Department of Ophthalmology, David Geffen School of Medicine at UCLA, 100 Stein Plaza, Los Angeles, CA 90095 USA; 2 Department of Microbiology Immunology and Molecular Genetics, Los Angeles, CA USA; 3 Eli and Edythe Broad Center of Regenerative Medicine and Stem Cell Research at UCLA, Los Angeles, CA USA; 4 Jonsson Comprehensive Cancer Center, Los Angeles, CA USA; 5 Department of Molecular Cell and Developmental Biology, Los Angeles, CA USA; 6 Department of Pediatrics, David Geffen School of Medicine, Los Angeles, CA USA; 7 Department of Population Sciences, City of Hope National Medical Center, Duarte, CA USA; 8 Department of Medicine, David Geffen School of Medicine, Los Angeles, CA USA; 9 Department of Neurobiology, David Geffen School of Medicine, Los Angeles, CA USA; 10 Molecular Biology Institute, Los Angeles, CA USA; 11 Brain Research Institute, University of California, Los Angeles, CA USA

**Correction to: Stem Cell Res Ther (2017) 8:217**

**https://doi.org/10.1186/s13287-017-0652-9**

The original article [1] contains an error in the legend of Fig. 5 whereby the descriptions for panels 5d and 5e are incorrect; as such, the corrected legend can be viewed below with its respective figure images.

**Fig. 5:**
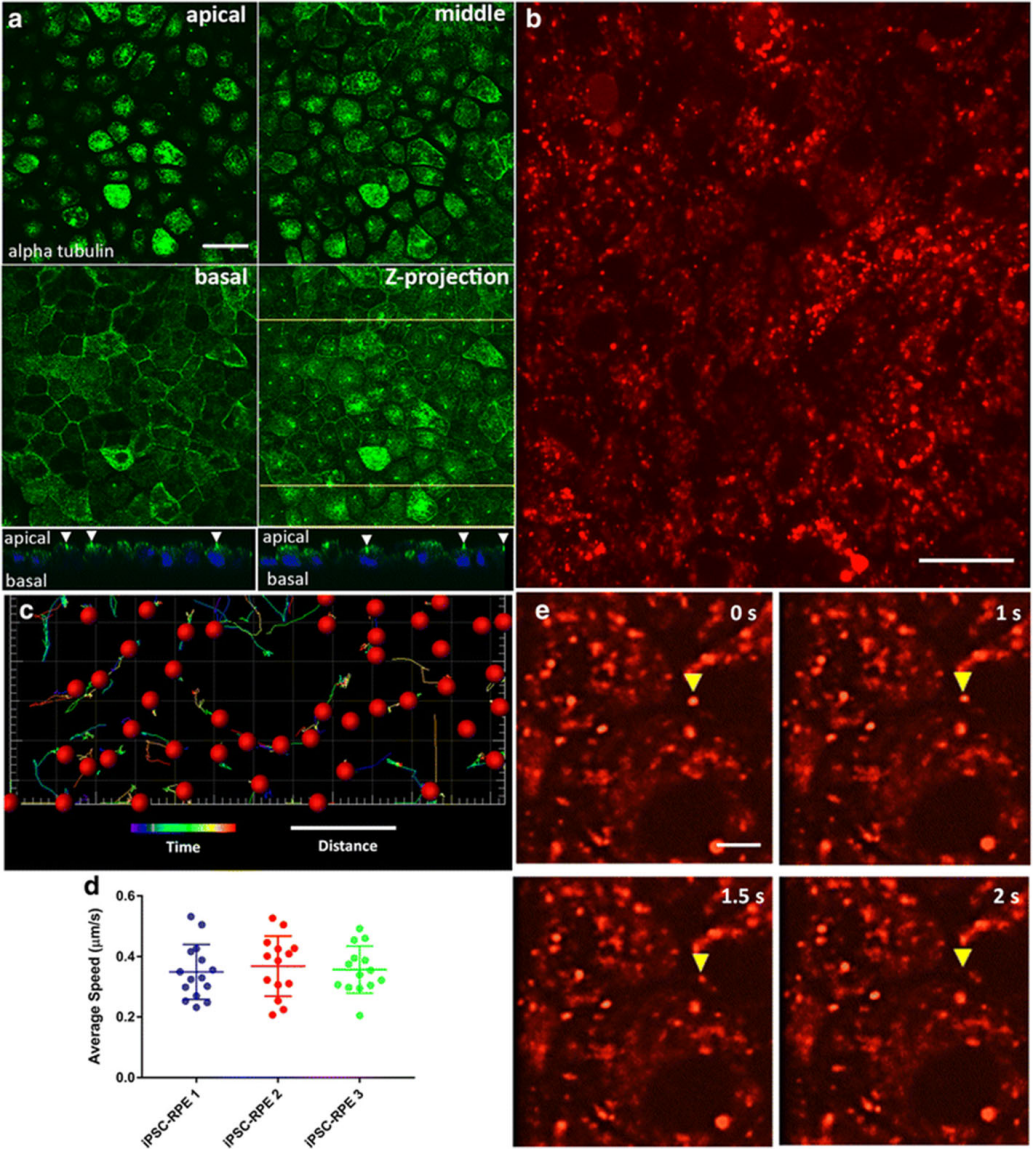
Microtubule organization and trafficking of endolysosomes in iPSC-RPE cells. **a** Microtubule organization in the iPSC-RPE cell bodies illustrated by immunostaining of alpha tubulin. Single-plane confocal microscopy images (2-μm apart) represent the apical and basal regions of the cell bodies, plus one plane in between (middle). The apical region of the cells is dominated by horizontal microtubules while the basal region is dominated by vertical microtubules. A z projection of the three panels is shown in the fourth panel. Below are images in two z planes at the yellow lines in the z-projection image, showing primary cilia (indicated by white arrowheads) emanating from the apical surface of the RPE cells. **b** Still image from a movie of iPSC-RPE cells that were incubated with red LysoTracker to label endolysosomes (see Additional file 4 for a similar movie). **c** Trajectory and movement analysis of a population of endolysosomes, using a spots and tracks analysis (Imaris), following movie acquisition over a 25-s interval. The tracks represent the trajectories of the organelles, while their colors are indicative of how far (in terms of time) they are with respect to the 25-s movie, with cool colors being closer to the beginning of the movie, and hot colors being closer to the end of the movie. **d** The average speed of endolysosomes was determined by analyzing the tracks of the organelles, and was found to be similar among the RPE cells derived from three independent iPSC lines. **e** Time-lapse images from a movie showing vertical movement of a labeled organelle (yellow arrowhead). Each panel represents the same z plane at different times. The organelle moves out of the plane after 2 s, indicating that it is traversing different z planes. Scale bars: **a**, **b**, 20 μm; **c**, 25 s (time), 5 μm (distance); **e**, 5 μm. iPSC induced pluripotent stem cell, RPE retinal pigment epithelium

## References

[1] Hazim RA (2017). Differentiation of RPE cells from integration-free iPS cells and their cell biological characterization. Stem Cell Res Ther.

