# Supplementary figures and images for: Differentiation of RPE cells from integration-free iPS cells and their cell biological characterization
Source: Stem Cell Res Ther. 2017 Oct 2;8:217. doi: 10.1186/s13287-017-0652-9 (PMC5625837; doi:10.1186/s13287-017-0652-9)

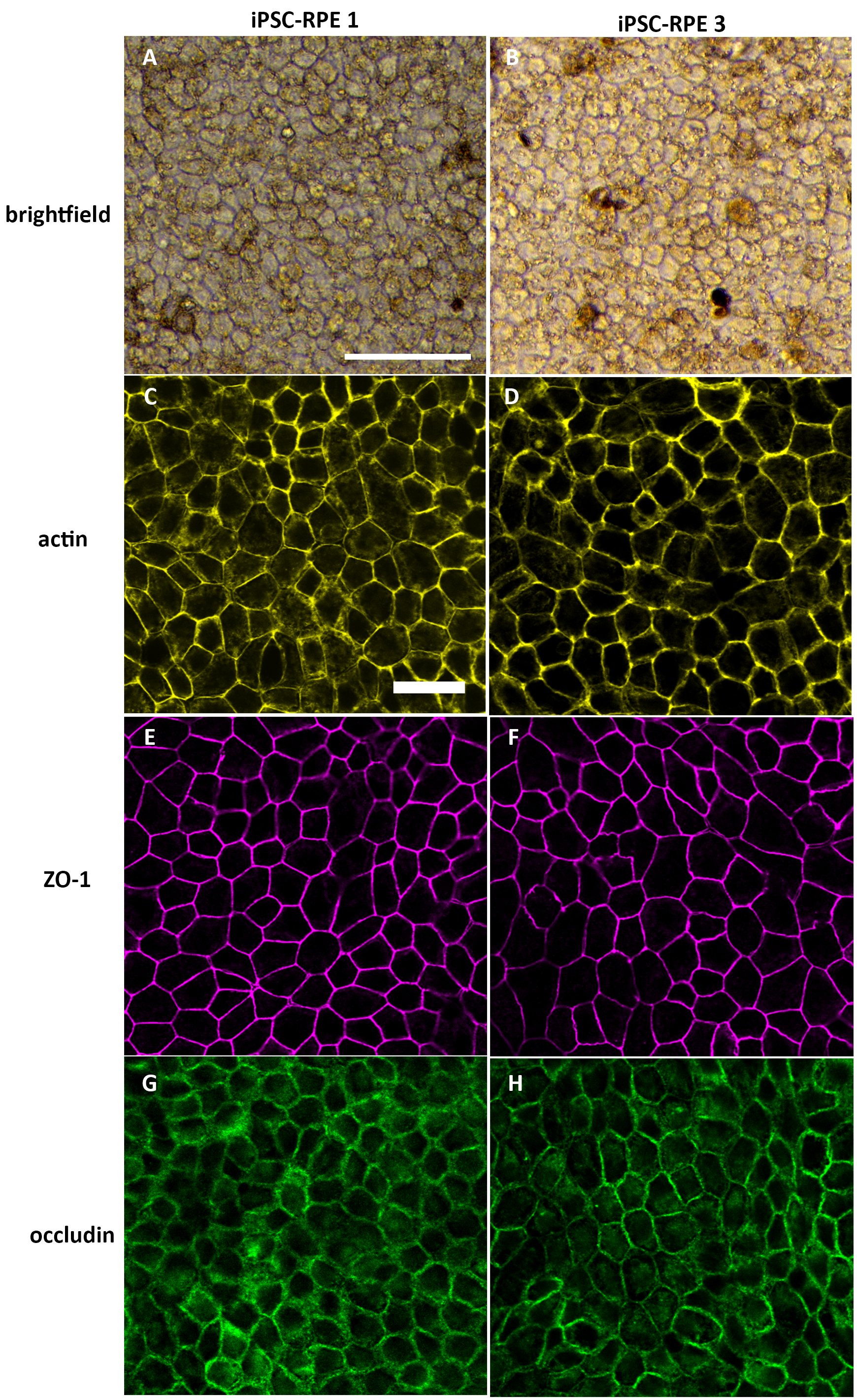

Supplement: Supplementary file 1 — Is a figure showing (A, B) brightfield micrographs of iPSC-RPE 1 (A) and iPSC-RPE 3 (B), illustrating the pigmentation and cobblestone morphology of the cells; (C, D) phalloidin labeling of iPSC-RPE 1 (C) and iPSC-RPE 3 (D), illustrating the cortical arrangement of actin filaments in the cells; (E–H) Immunofluorescence micrographs, illustrating expression of the tight junction proteins, ZO-1 (E, F) and occludin (G, H), in iPSC-RPE 1 and 3. Scale bars: A, B, 60 μm; C–H, 20 μm. (TIF 5893 kb) [file 13287_2017_652_MOESM1_ESM.tif]

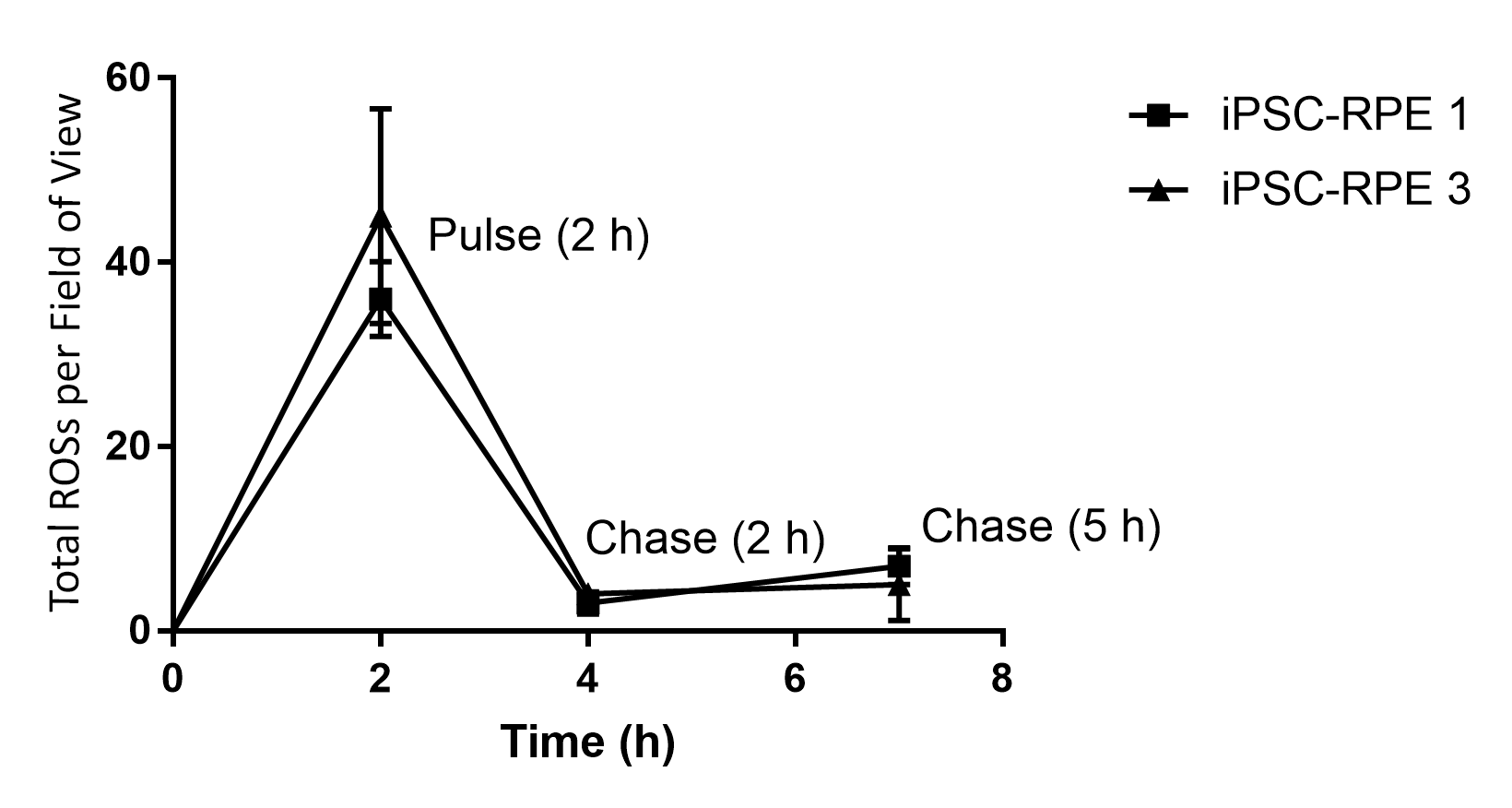

Supplement: Supplementary file 2 — Is a figure showing quantification of phagocytosis in iPSC-RPE 1 and 3 exposed to porcine POSs for 2 h (pulse), washed extensively to remove unbound POSs, and then allowed either a 2-h or 5-h chase period to ingest and degrade the POSs. Graph shows the total number of ROSs quantified from confluent fields of view after the pulse and the two separate chase periods. Data represent mean ± SD. (TIF 89 kb) [file 13287_2017_652_MOESM2_ESM.tif]

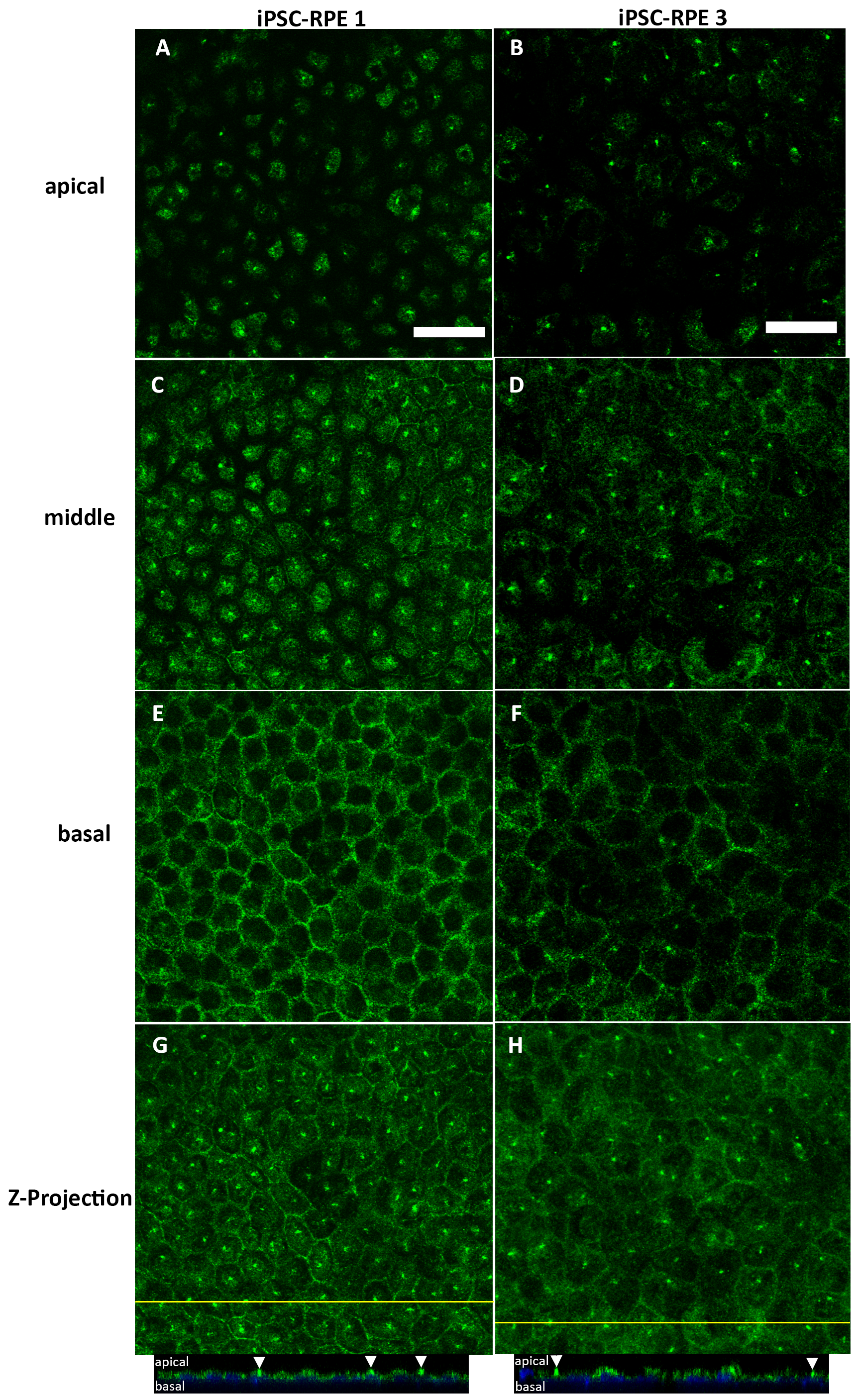

Supplement: Supplementary file 3 — Is a figure showing alpha tubulin labeling in iPSC-RPE 1 (A, C, E, G) and iPSC-RPE 3 (B, D, F, H) showing the arrangement of microtubules in an apical region (A, B), middle region (C, D), and basal region (E, F) of the cells. The apical region is dominated by horizontally-oriented microtubules whereas the basal region consists mainly of vertically-oriented microtubules. (G, H) z projections; z planes at the locations of the yellow lines illustrating the presence of primary cilia (indicated by white arrowheads) on the apical surface of the iPSC-RPE cells. Scale bars: 20 μm. (TIF 4278 kb) [file 13287_2017_652_MOESM3_ESM.tif]
